# Epoxy Composites with Reduced Graphene Oxide–Cellulose Nanofiber Hybrid Filler and Their Application in Concrete Strain and Crack Monitoring

**DOI:** 10.3390/s19183963

**Published:** 2019-09-13

**Authors:** Zhiqiang Wu, Jun Wei, Rongzhen Dong, Hao Chen

**Affiliations:** 1 School of Civil Engineering, Central South University, Changsha 410075, China; 2 China Construction Second Engineering Bureau Co., Ltd., Beijing 100071, China

**Keywords:** nanotechnology, polymer composite, piezoresistivity, flexible sensor, concrete health monitoring

## Abstract

Advances in nanotechnology have provided approaches for the fabrication of new composite materials for sensing. Flexible sensors can make up for the shortcomings of traditional strain sensors in monitoring the surface strain and cracks of concrete structures. Using reduced graphene oxide (RGO) as a conductive filler, cellulose nanofiber (CNF) as a dispersant and structural skeleton, and waterborne epoxy (WEP) as a polymer matrix, a flexible composite material with piezoresistive effect was prepared by the solution blending and solvent evaporation method. The mechanical, electrical, and electromechanical properties of the composite were investigated. The results show that CNF can significantly improve the dispersion of RGO in the WEP matrix and help to form stable reinforcing and conductive networks, leading to great changes in the mechanical properties and resistivity of the composite. The composite film can withstand large deformations (>55% strain), and the resistance change rate demonstrates a high sensitivity to mechanical strain with a gauge factor of 34–71. Within a 4% strain range, the piezoresistive property of the composite is stable with good linearity and repeatability. The performance of the flexible film sensor made of the composite is tested and it can monitor the strain and crack of the concrete surface well.

## 1. Introduction

Concrete is the most widely used material in civil engineering structures [1]. The service life of a concrete structure lasts for several decades or even longer. In long-term processes, the combined actions of multiple factors, such as the load effect, environmental erosion, and material aging, will lead to damage accumulation and resistance attenuation [2,3,4]. Deformation and cracking are the most direct phenomena of structural damage [5,6]. If the deformation and cracking cannot be effectively monitored and located, the structure cannot be repaired and strengthened in time, which will affect the service performance of the structure, and could even lead to catastrophic accidents. Therefore, structural health monitoring is necessary to determine the stress and strain states of key positions and to evaluate the safety and reliability of the structure [7].

A strain sensor is a functional device for measuring the strain produced by forces and deformation. It has a wide range of applications in structural damage detection and health monitoring [8,9]. Traditional resistance strain sensors are mainly made of metal or semiconductor materials based on the piezoresistive effect. The characteristics of metal and semiconductor materials limit the performance of the sensor, resulting in some defects, such as a small range, low sensitivity, poor flexibility, and susceptibility to damage. In addition, the application of traditional strain sensors in large strain monitoring and structures with complex surfaces is increasingly unable to meet the needs of structural health monitoring. So, it is necessary to develop a new strain sensor with a wide range, high sensitivity, good durability, and low cost [10].

Nanomaterials with unique structures and properties can be used to enhance and modify cement-based materials or polymers, which provides a method for the preparation of new sensors [11,12,13,14]. Nano carbon black [15,16,17], carbon nanotube [18,19,20,21,22], and hybrid filler [23,24,25] are often used as conductive fillers to synthetize composites with piezoresistive properties. However, cement composites are quasi-brittle materials, which are difficult to make into thin films and are seldom used for structural surface monitoring. Usually, they are made into blocks and used as embedded sensors for interior monitoring. Polymers have excellent mechanical properties and good plasticity, which are also inexpensive and easy processing. The piezoresistive composite obtained by polymer doped with conductive filler is an ideal material for flexible strain sensors. It can overcome the shortcomings of traditional strain sensors. Using the polymer-based composite, thin film sensors can be made to monitor the strain and cracking on the structural surface. 

In recent years, graphene, a two-dimensional nanomaterial with excellent mechanical and electrical properties, has attracted significant attention and has been studied extensively [26,27,28,29]. Compared with one-dimensional nanomaterials such as carbon nanotubes, graphene has a larger specific surface area, which is more conducive to the transmission of electrons. When uniformly dispersed in the polymer, it easily forms a stable conductive network with a lower filler content. Additionally, the composite has better conductivity and repeatability. However, similar to carbon black and carbon nanotube, graphene is hydrophobic and oleophobic. So, graphene is not easily dispersed in inorganic or organic solvents, which limits its application. Physical and chemical methods are used to improve the dispersion of graphene [30]. Physical methods mainly include mechanical stirring and ultrasonic dispersion. The dispersion solution obtained often has a low concentration of dispersed filler and a short duration of being in a uniform dispersion state. Graphene rapidly reunites under π-π action after physical dispersion. Chemical methods usually require the use of toxic and hazard reagents to modify graphene, which is not environmentally friendly. Therefore, a new dispersion method is needed.

Cellulose is the most abundant natural macromolecule material with degradable and renewable characteristics, which widely exists in wood, cotton, and some bacteria. Cellulose nanofiber (CNF) is a kind of nanofiber that is separated from cellulose. It not only has a large specific surface area and excellent mechanical properties but also has the characteristics of high reactivity and strong hydrophilicity [31,32]. Hydrophilicity enables CNF to form a uniform and stable suspension in water, and high reactivity makes CNF easy to bind with graphene. Thus, CNF can be used as a carrier of graphene to improve its dispersion in aqueous solution. The application of CNF in the preparation of flexible conductive materials can reduce costs and environmental pollution. The excellent mechanical properties of CNF can also increase the flexibility of the composites.

There are many kinds of polymers, and the selection is a key point. Waterborne epoxy (WEP) is used as the polymer matrix. WEP emulsion can dissolve in water and fully mix with aqueous suspensions of nanofillers so that they are evenly dispersed in the WEP matrix. In addition, WEP has excellent mechanical properties which are not easy to damage and good plasticity which is easy to film. WEP is also inexpensive. After curing and film formation, the composite film has good flexibility, neither too soft like rubber polymers, nor brittle like pure epoxy resin. 

In this study, first, a flexible composite material with a piezoresistive effect is prepared using reduced graphene oxide (RGO) as the conductive filler, CNF as the dispersant and structural skeleton, and WEP as the polymer matrix. Second, the mechanical, electrical, and electromechanical properties of the composite are investigated. The results show that CNF solves the dispersion problem of RGO well and helps to form stable conductive networks in the WEP matrix. The hybrid filler of two-dimensional RGO and one-dimensional CNF has a great influence on the mechanical and electrical properties of the composite. The composite films can withstand large deformations, and the resistance change rate demonstrates high sensitivity to mechanical strain. Within a 4% strain range, the piezoresistive property of the composite is stable with good linearity and repeatability. Finally, the performance of the flexible film sensor made of the composite is tested, and it can monitor the strain and crack of the concrete surface well.

## 2. Materials and Methods

RGO (purity > 98 wt%, thickness 1–5 nm, flake size 0.5–5 μm), CNF (purity > 99 wt%, diameter 4–10 nm, length 1–3 μm), WEP (white emulsion, solid content 50 ± 2 wt%, product number: F0704), and a curing agent are commercially available and were used in the fabrication of the composite.

**Figure 1:**
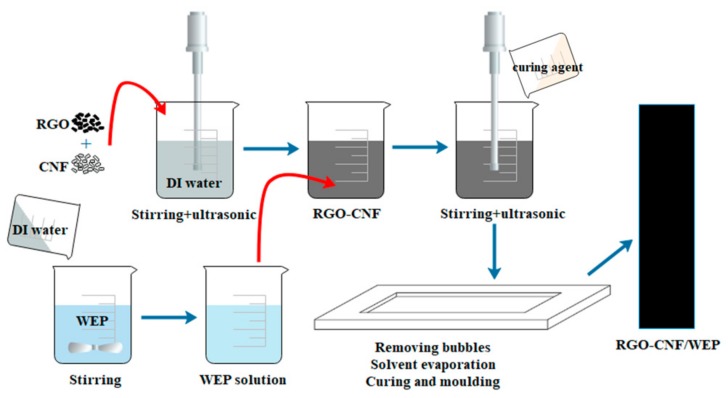
Fabrication process of the composite.

The composite was prepared by an environment-friendly solution blending and solvent evaporation method. The fabrication process is briefly shown in Figure 1. Since the WEP emulsion has high viscosity, it is not conducive to the dispersion of RGO. So, the WEP emulsion was mixed with deionized (DI) water and magnetically stirred at a speed of 1000 r/min for 5 min at first. This yielded an aqueous solution of WEP with low viscosity. Due to the hydrophobicity of RGO, it would agglomerate when added in water. Hydrophilic CNF was selected as a carrier to help the dispersion of RGO. In the second step, RGO and CNF powders were added to DI water, magnetically stirred at a speed of 2000 r/min for 5 min and then ultrasonically treated at 200 W for 30 min. Then, the obtained suspension solution of RGO-CNF was added into the WEP aqueous solution with magnetic stirring at a speed of 1000 r/min for 5 min, followed by ultrasonic dispersion at 200 W for 1 h. A solution with RGO-CNF uniformly dispersed in WEP was obtained. Then, curing agent was added at a weight ratio of 1:2. After 5 min of magnetic stirring at a speed of 1000 r/min, it was placed in the vacuum box for 30 min to remove the bubbles and subsequently put in the oven to complete curing at 40 °C for 3 h and at 60 °C for 24 h. The choice of curing temperature is key to the formation of a stable conductive network. If the temperature is high and the water evaporates too quickly, the network between graphene flakes will be destroyed, thus affecting the electrical properties of the composite. The amount of RGO in the RGO-CNF/WEP composites was 0.4%, 0.8%, 1.2%, and 1.6% by weight of WEP, respectively. The content of CNF was half that of RGO.

## 3. Results and Discussion

### 3.1. Dispersion of RGO

The dispersion state of conductive fillers in polymer matrix determines the electrical properties of the composite. RGO is added into the WEP matrix in two steps, and the key first step is to obtain a uniformly dispersed RGO aqueous suspension. Due to the few surface group, low chemical activity, and high specific surface area, an agglomeration phenomenon occurs when RGO is added to water. Although RGO can be dispersed under ultrasonic treatment, the concentration of dispersed RGO is low and the duration of the dispersion state is short. RGO rapidly reunites under π-π action. There is a large number of hydrophilic hydroxyl groups on the surface of CNF, which enables CNF to form a stable and uniform aqueous suspension under rapid agitation. Some unreduced hydroxyl and carboxyl groups are distributed on the surface of RGO. They can interact with the hydroxyl groups on the surface of CNF by hydrogen bonding. With CNF as the carrier, RGO can be dispersed uniformly and stably in water by mechanical stirring and ultrasonic treatment. The obtained aqueous suspension of RGO-CNF is mixed with WEP to disperse RGO into the composite.

**Figure 2:**
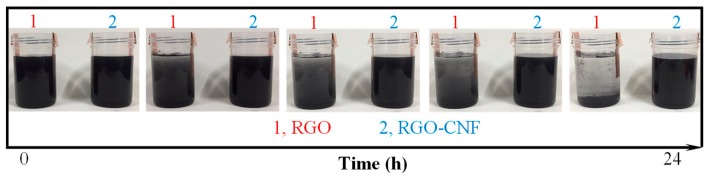
Comparison of graphene oxide (RGO) and RGO–cellulose nanofiber (CNF) aqueous suspensions with static treatment for 24 h.

**Figure 3:**
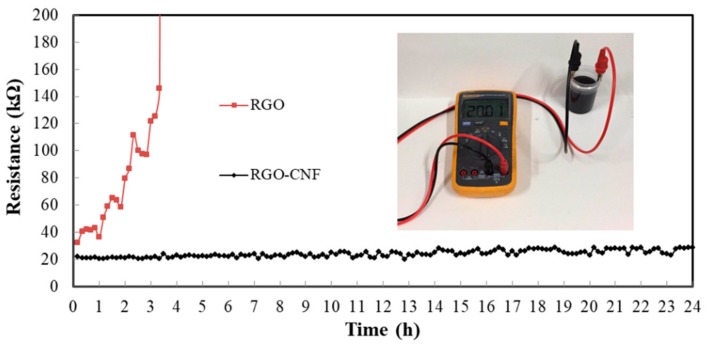
The changes in resistance of two suspensions over time.

Figure 2 is a comparison of RGO and RGO-CNF aqueous suspensions with a static treatment for 24 h. It can be seen that the RGO suspension has obvious agglomeration and precipitation over time, while the RGO-CNF composite suspension still maintains a uniform dispersion state. Figure 3 shows the changes in resistance of two suspensions over time. The resistance of RGO suspension increases with time, while the resistance of the RGO-CNF suspension is relatively stable. These both indicate that CNF can effectively carry and assist RGO to disperse in water and form a uniform and stable suspension. This method can also be used for the dispersion of carbon nanotubes, which are cylindrical structures made up of curled graphene.

### 3.2. Mechanical and Electrical Properties

The mechanical properties were tested using a tensile instrument (HP-500, LANB instrument, Guangzhou, China), and the electrical properties were tested using a digital multimeter (F15B+, Fluke, Shanghai, China) and an electrochemical workstation (CHI650E, Shanghai, China). The specimens were cut into rectangular shapes (60 mm × 10 mm × 0.5 mm), and at least three effective specimens were tested for each sample.

**Figure 4:**
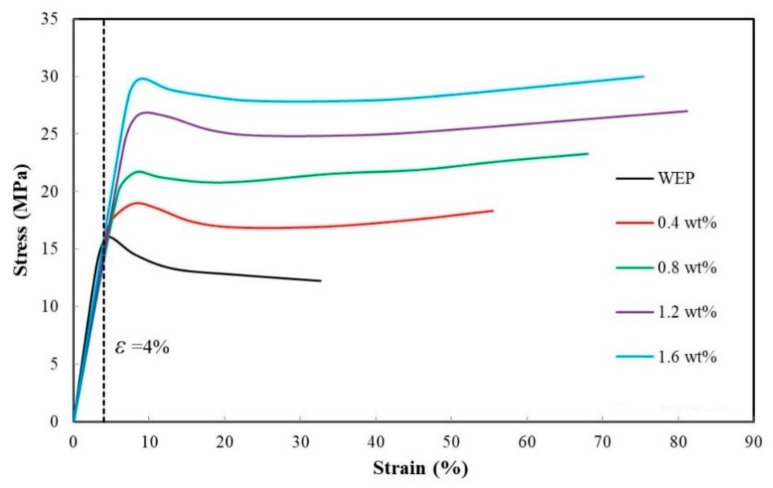
The stress–strain curves of the composite.

Figure 4 shows the stress–strain curves of the composite. It indicates that the addition of RGO and CNF significantly improves the mechanical properties of WEP. Thanks to the high strength of RGO, the tensile strength of the composite rises with the increase in the RGO content. CNF has good flexibility. With the increase in the CNF content, the elastic modulus of the composite tends to decrease slightly, which makes the composite more flexible. Similar to the role of steel bar in reinforced concrete, CNF acts as the structural skeleton of the composite. This may be the reason why the composite can present ductile failure. Another reason for the existence of the yield point is that the emulsifier in WEP acts as a plasticizer in the composite film.

Table 1 shows the resistivity and conductivity of the composite. The resistivity decreases with the increase in RGO content. With the help of CNF, the conductive filler RGO is evenly dispersed in the WEP matrix. When the distance between RGO flakes is small enough, a tunnel effect occurs and conductive paths form in the composite. This makes the resistivity of WEP decrease significantly and the composite becomes a conductor. The percolation threshold is the critical value of RGO content that changes the composite from an insulator to a conductor. It can be inferred that the percolation threshold of the composite is less than 0.4 wt%.

The improvements in the mechanical and electrical properties of the RGO-CNF/WEP composite mainly depend on the binding state of RGO and CNF, the dispersion level of RGO-CNF in WEP and the combination situation of RGO-CNF with WEP. As a green and renewable one-dimensional nanomaterial, CNF acts not only as a dispersant but also as a structural skeleton in the composite. On the one hand, CNF combines with RGO through hydrogen bonds. Benefiting from the good hydrophilicity of CNF, the overlapping CNF can carry RGO to disperse evenly in the WEP matrix. It helps to form a stable and continuous conductive network of RGO in the WEP matrix. Moreover, owing to the excellent mechanical properties of RGO and CNF, a cross-linking reinforced network is also constructed, which significantly improves the mechanical properties of the composite. On the other hand, the good compatibility of CNF ensures close integration with WEP. CNF serves as a bridge between RGO and WEP.

At an RGO content of 0.4 wt% to 1.6 wt%, the tensile strength and conductivity (reciprocal of resistivity) of the composite show increasing trends. However, if RGO and CNF are added excessively, the density of the fillers increases significantly and the distance between flakes becomes small enough to generate an agglomeration due to Van der Waal forces. CNF cannot carry redundant RGO to be uniformly dispersed into the WER matrix, which destroys the balance among the three components in the composite. The redundant RGO and CNF agglomerate in the WER matrix, resulting in a stress concentration which decreases the mechanical properties of the composite, also affecting the electrical properties. In addition, the cluster of fillers will decrease the bonding with the WEP matrix.

### 3.3. Electromechanical Properties

The resistivity of the composite with an RGO content of 0.4 wt% is relatively high and not conductive to electrical signal measurement. So, the samples of the RGO-CNF/WEP composite with RGO contents of 0.8 wt%, 1.2 wt%, and 1.6 wt% were tested. For comparison, a sample of the RGO/WEP composite with an RGO content of 0.8 wt% was also tested. When the composite material is deformed by external force, its internal conductive network also deforms and the distance between RGO flakes changes, which makes the composite resistance change. The phenomenon of resistance changing with strain is a piezoresistive effect. The gauge factor (GF) was used to evaluate the strain sensing performance of the composite films. It connects resistance change rate (RCR) with strain, as shown in Equation (1):

(1) $$GF=\frac{\left(R-R_{0}\right)/R_{0}}{\varepsilon }=\frac{\Delta R/R_{0}}{\varepsilon }=\frac{RCR}{\varepsilon }$$

where *ε* is the strain of the composite film, *R*_0_ is the initial resistance, and *R* is the resistance at strain *ε*.

**Figure 5:**
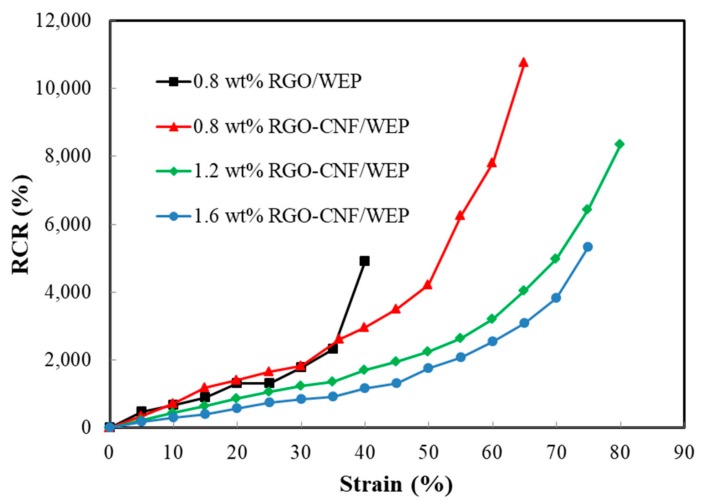
The correlation of the resistance change rate (RCR) and strain of four film samples.

**Figure 6:**
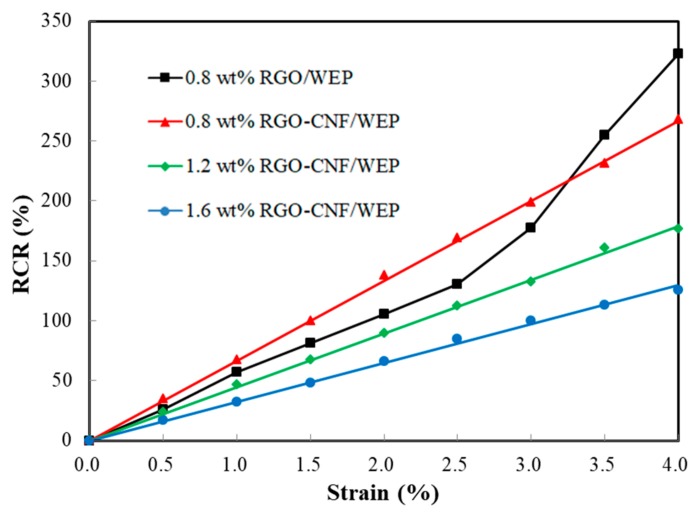
The correlation of RCR and strain within a 4% strain range.

Figure 5 shows the correlation between RCR and strain of four film samples. The curves indicate that the RCR has a good linear relationship with the strain in about a 10% strain range. As the strain increasing further, the RCR shows an approximately exponential growth trend. From Figure 4, it can be seen that the composites are in the elastic deformation stage when the strain is less than 4%. So, in particular, the correlation of RCR and strain within 4% the strain range is plotted in Figure 6. The curve linearity of the RGO-CNF/WEP composite is obviously better than that of the RGO/WEP composite. The slope of the curve equals the GF of the film sample, and the RGO-CNF/WEP composite is larger than the RGO/WEP composite with an RGO content of 0.8 wt%. It can be concluded that the addition of CNF can significantly improve the piezoresistive properties of the composites. The GFs of the three RGO-CNF/WEP composite samples were found to be 71, 47, and 34, respectively. They were all much larger than that of the traditional strain gauge (~2). The GF increased as the RGO content decreased. In other words, film with higher resistivity is more sensitive to strain change.

**Figure 7:**
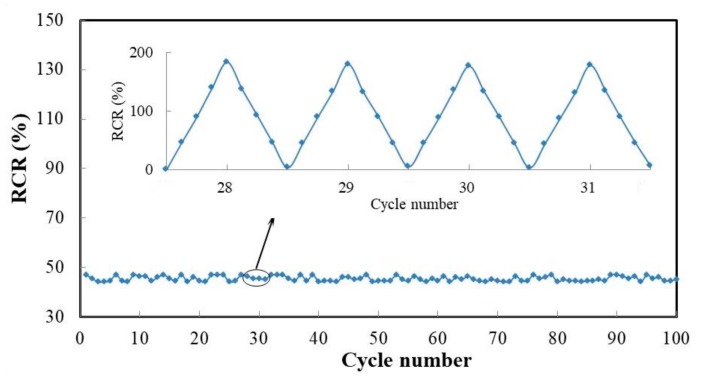
Gauge factor (GF) of sample 1 during 100 cycles of tension within 4% strain region.

The repeated test of sample with 1.2 wt% RGO was carried out, and its GF was almost unchanged during 100 cycles of tension within the 4% strain region, as shown in Figure 7. It can be seen that the piezoresistive properties of the composite film were stable with good linearity and repeatability. The slight change in GF under cyclic tension can be explained by the low energy dissipation of sliding friction of graphene flakes [29]. The 4% strain is obviously larger than the ultimate tensile strain of concrete, which means the composite film can be used for multiple measurement of concrete strain before cracking. In addition, the GF of the composite film can be adjusted by adjusting the contents of RGO and CNF according to specific requirements, for example, when measuring low strain to adjust to high sensitivity and high strain to low sensitivity [33].

The conductive network of the composite is formed by uniformly dispersed RGO, and the piezoresistivity of the composite is mainly due to changes in the distance between RGO flakes. The piezoresistive model can be described by the following equation [34,35]:

(2) $$\mathrm{GF}=\frac{\Delta R/R_{0}}{\varepsilon }=\frac{\left(1+\varepsilon \right)\exp\left[\left(\gamma s_{0}+A\right)\varepsilon +B\varepsilon^{2}\right]-1}{\varepsilon }$$

When the strain *ε* is small, the GF can be expressed as follows:

(3) $$\begin{array}{l} \mathrm{GF}=\underset{\varepsilon \to 0^{+}}{\lim}\frac{\Delta R/R_{0}}{\varepsilon }\approx \underset{\varepsilon \to 0^{+}}{\lim}\frac{\exp\left[(\gamma s_{0}+A)\varepsilon +B\varepsilon^{2}\right]-1}{\varepsilon }\\ \approx \underset{\varepsilon \to 0^{+}}{\lim}\frac{\left[\left(\gamma s_{0}+A\right)+2B\varepsilon \right]\exp\left[\left(\gamma s_{0}+A\right)\varepsilon +B\varepsilon^{2}\right]}{1}\\ \approx \gamma s_{0}+A \end{array}$$

where *A*, *B*, and *γ* are constants related to the conductive filler, and *s*_0_ is the initial distance between RGO flakes.

Equations (2) and (3) show that the RCR of composite films is proportional to the deformation degree in the small strain region, and as the tension degree increases, the RCR shows an exponential trend in the large strain region, which is consistent with the measured results. Within the linear range, the GF is approximately equal to (*γs*_0_ *+ A*), which is related to the type and initial distance *s*_0_ of the conductive filler. The initial distance *s*_0_ is inversely proportional to the content of the conductive filler. So, on the premise that the types of conductive fillers are determined and dispersed uniformly, the sensitivity of composite films can be improved by decreasing the content of the conductive filler. However, when the content of the conductive filler is low, the resistance of the film is large, which is not conducive to the acquisition of electrical signals in practical applications. The large content will affect the mechanical properties of the composites. The excellent properties of the polymer matrix will be greatly lost, and the sensitivity coefficient of the film will be reduced. Therefore, the choice of filler concentration should take both sides into account.

### 3.4. Strain and Crack Monitoring on a Concrete Surface

**Figure 8:**
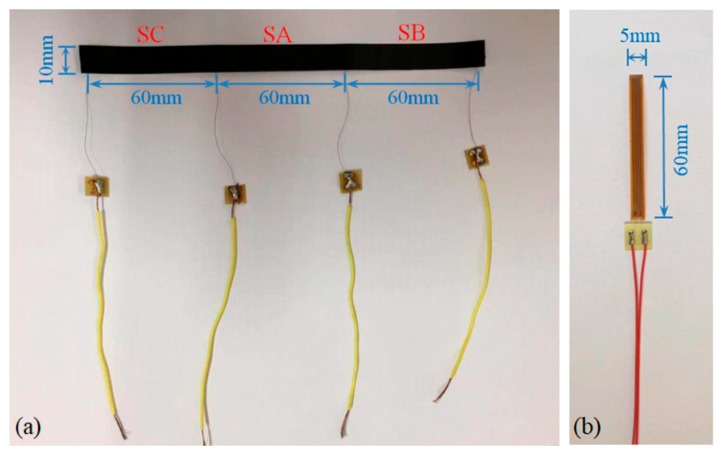
(**a**) A multi-electrode film sensor; (**b**) a traditional resistance strain gauge.

**Figure 9:**
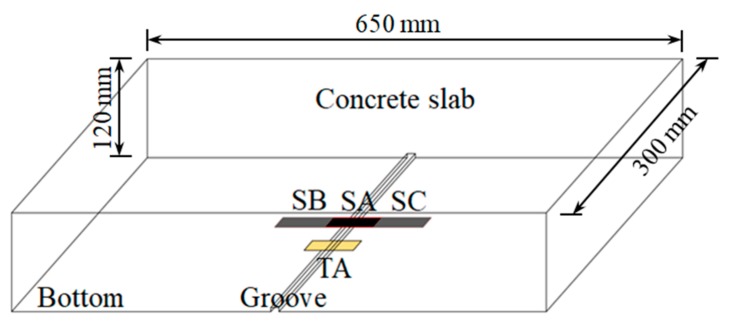
The positions of the film sensor and resistance strain gauge.

**Figure 10:**
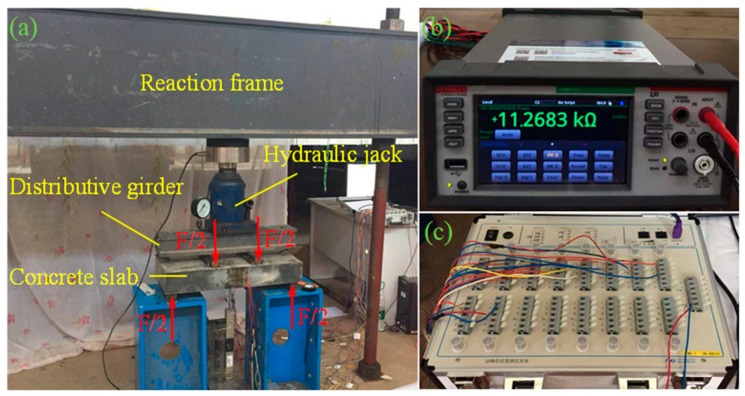
Layout of the experimental setup: (**a**) four-point loading chart; (**b**) source meter; (**c**) static resistance strain indicator.

Due to the low tensile strength, concrete is easy to crack in the tension zone. A traditional resistance strain gauge can only measure the strain of concrete before cracking because of its small measuring range and cannot monitor the situation after cracking. The above analysis shows that the composite film has excellent piezoresistive properties and can withstand large deformations, so it may be used to monitor concrete cracks. A multi-electrode film sensor was fabricated as shown in Figure 8a. The composite film was divided into three test sections, SA, SB, and SC, by four electrode tabs. The electrode tabs were made of copper foil and embedded in the film. In order to verify the sensing properties of the composite film to concrete strain and crack, a concrete slab with a size of 650 mm × 300 mm × 120 mm was poured. The slab was equipped with a small amount of reinforcement so that it could continue to bear load after concrete cracking. The multi-electrode film sensor was bonded to the slab by structural adhesive. As shown in Figure 9, the middle section of the film sensor was located in the middle of the bottom of the slab, where a groove with a width of 2 mm and a depth of 5 mm was reserved for the crack to first appear, and a traditional resistance strain gauge (TA, as shown in Figure 8b) was pasted parallel to SA. Figure 10 shows the layout of the experimental setup. The load was applied by a hydraulic jack (YZF500, Haili Machinery Manufacturing Co., Ltd., Taizhou, China) through a reaction frame, and four-point loading was used. The strains of SA, SB, SC, and TA were collected using a static resistance strain indicator (UT7121Y, Youtai Electronic Technology Co., Ltd., Wuhan, China). The resistances of SA, SB, and SC were measured using a 6.5-digit source meter (DMM6500, Keithley Instruments, Cleveland, OH, USA).

**Figure 11:**
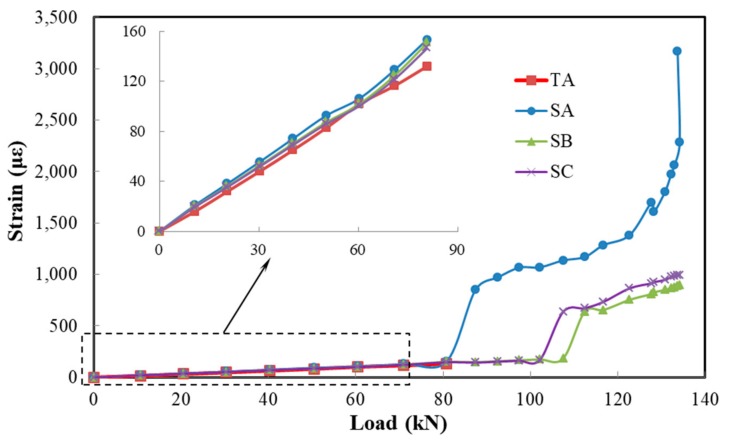
Strains of composite film sensors (SA, SB, and SC) and traditional resistance strain gauge (TA) during loading.

Figure 11 shows the strains of SA, SB, SC, and TA during loading. As the load increases, the strain of concrete in tension zone increases gradually. When the load reaches 80.7 kN, the strain of TA is 132 microstrains. This means that the average strain of concrete in the length range of TA is also 132 microstrains. When the load reaches 87.5 kN, TA loses its signal, but the strain value of SA changes abruptly. Observations of the concrete near SA and TA show that a crack appears. Although the average strain of concrete at this time is much less than the ultimate tensile strain of TA (20,000 microstrains), the strain of the concrete in the local area near the crack exceeds the ultimate tensile strain of TA. So, TA is broken and does not work after the load of 87.5 kN. However, SA can still work because of the good deformability of the composite film. As the load increases, the strain value of SA continues to increase. Before the concrete cracks, the measured strain of SA is close to that of TA, which indicates the feasibility and accuracy of measuring the concrete strain using the composite film. There is also a slight difference between both. This can be attributed to the following three possible reasons: (i) the flatness of the slab bottom may be insufficient, and bubbles would be introduced when sensors are not pasted well, affecting the monitoring results, (ii) the composite film is much thicker than the traditional resistance strain gauge, which makes the strain transfer different, and (iii) the Poisson’s ratio differs between the composite material and concrete. This results in the GF of the isolated composite film measured by stretching having a tiny difference to the actual GF when bonded to concrete. In addition, SA, SB and SC are all in a pure bending section, but the strain of SA is a little larger than the strain of SB and SC before the concrete cracks. This is because that the groove decreases the inertial moment of the middle section. After concrete cracking, the traditional resistance strain gauge is damaged, while the composite film can still monitor the signal. Subsequently, the SB and SC strains also increase abruptly, and cracks occur at the corresponding locations. However, if the strain after concrete cracking exceeds 4%, the measured strain is not the actual value. This is due to the large strain of the film after cracking, which results in a great change in the GF of the film, but the initial input of GF is constant. In practical applications, it is necessary to modify the monitoring value after cracking.

**Figure 12:**
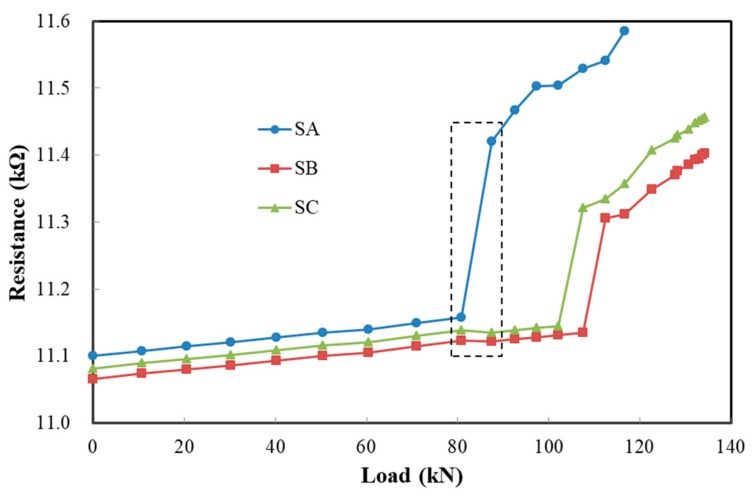
The resistance changes in SA, SB, and SC during loading.

Figure 12 shows the resistance changes of SA, SB and SC during loading. Before concrete cracking, their resistances all increase slowly with the load. As shown in the dotted box, after the appearance of the first crack in the middle part, the resistance of SA changes abruptly, while the resistances of SB and SC in the non-cracked region do not. On the contrary, a slight decrease in the resistances of SB and SC can be observed. This is due to stress redistribution after concrete cracking. After cracking, the resistances of SA, SB and SC continue to increase slowly with the load. Subsequently, two sudden changes in the resistances of SB and SC emerge, which indicates that there are two cracks at the locations of SB and SC. The locations of the cracks can be determined by the position of the film with the resistance changing abruptly. If real-time monitoring is used, the cracking time can also be determined. The width of the crack can be calculated by the magnitude of the abrupt resistance change according to Equation (2). Knowing the location and width can give an early warning about the crack. The above analysis shows that the composite film has a good monitoring performance for concrete strain and cracks.

### 3.5. Design of Composite Mesh Film Sensor and Its Application Outlook

**Figure 13:**
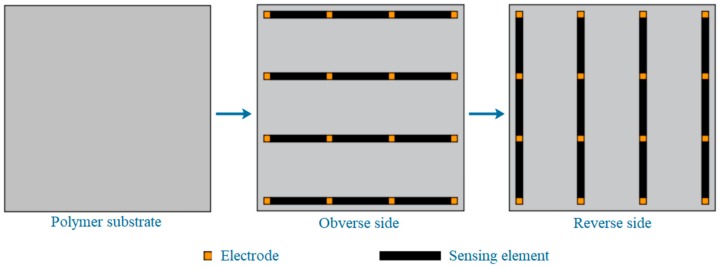
The composite mesh film sensor.

For some complex structures, it is sometimes necessary to monitor the deformation, especially cracks, in larger areas. However, the monitoring area of a rectangular composite film sensor (RCFS) is quite limited, and it is not economical and convenient to use multiple RCFSs for regional monitoring. The composite inherits the good plasticity of WEP. By using the screen-printing technique, multiple RCFSs can be integrated into a thin film, and a composite mesh film sensor (CMFS) is designed, as shown in Figure 13. Transverse sensing elements are printed on one side of a polymer film, longitudinal sensing elements are printed on the other side, and then two protective films are covered on both sides. According to the position of the sensing element (SE) with abrupt resistance change, the position of the crack is preliminarily determined. Further, if the midpoint coordinate of the SE is taken as the coordinate of the crack at this position, a series of points can be attained with the development of the crack. Then, the approximate trend of the crack can be obtained by multi-point fitting, and its accuracy may be related to the distribution density of the SE. The width of crack can also be obtained by Equation (2).

**Figure 14:**
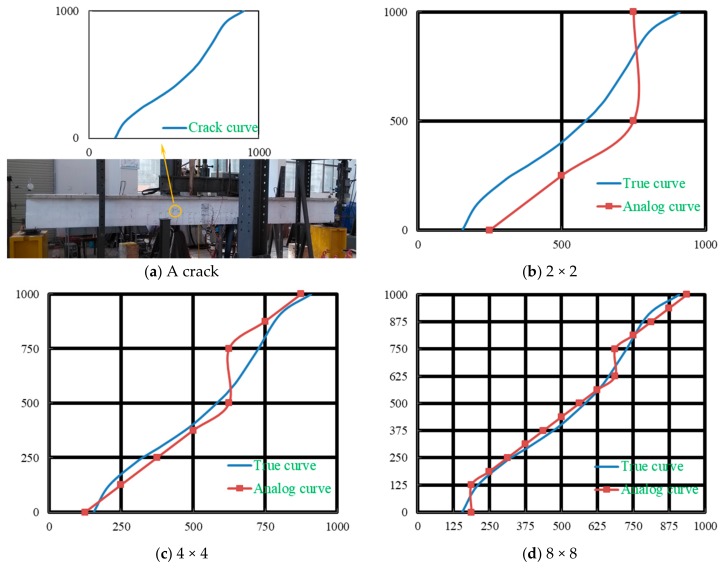
(**a**) A crack on the surface of a concrete beam; (**b**–**d**) Simulation results of the crack using composite mesh film sensors (CMFSs) with different sensing element (SE) distribution densities.

Figure 14a shows a crack on the surface of a concrete beam caused by fatigue load. If the crack area was monitored using CMFS before the crack occurred, crack trend may be obtained by the above method. Figure 14b–d shows simple simulation results of the crack trends monitoring using CMFSs with different SE distribution densities. It can be seen that the larger the distribution density is, the closer the analog curve is to the true curve. It is noteworthy that this applies only to the case in which there is only one crack in the monitoring area, and further discussion is needed when multiple cracks occur. The thickness of the film can be very small. When it is inconvenient to further improve the distribution density of SE, the multi-layer composite mesh film can be used to improve the monitoring accuracy. In addition, epoxy is often used as a reinforcement material. The composite prepared in this paper may be used as a reinforcement material, and at the same time, the structural performance after reinforcement can be monitored.

To achieve the above prospects, the filler content and preparation process of composite materials need to be adjusted for practical applications. For example, in order to accelerate the curing of the composite in screen printing, isopropanol can be used to replace part of the water in the solvent. Isopropanol is soluble in water and has good volatility. When volatilized, it takes away water quickly and accelerates curing. Additionally, in order to improve the strength of the composite, it is necessary to adjust the proportions of fillers.

## 4. Conclusions

In this paper, a novel sensing material of the RGO-CNF/WEP composite was prepared to monitor the strain and cracks on the surface of a concrete structure. With the help of CNF, a stably and uniformly dispersed aqueous suspension of RGO-CNF was obtained. This is an environmentally friendly method to fabricate composites with a well dispersed RGO conductive filler. CNF can effectively carry and assist RGO to disperse in the WEP matrix and help form stable three-dimensional reinforcing and conductive networks. 

The effect of the RGO-CNF hybrid filler on the properties of the composite was investigated and discussed. RGO-CNF uniformly dispersed in WEP has a great influence on the mechanical and electrical properties of the composite. The thin film made of the composite can withstand large deformations (>55% strain), and its elastic strain range (>4% strain) is much larger than the ultimate tensile strain of concrete. With the addition of RGO and CNF, the resistivity of the composite decreased significantly. When the contents of RGO and CNF were 1.6 wt% and 0.8 wt%, respectively, the resistivity was 0.925 Ω·m. The percolation threshold of the composite was less than 0.4 wt%. Compared with adding RGO only into WEP, the RGO-CNF/WEP composite showed excellent piezoresistive properties. Within the 4% strain range, the piezoresistive properties of the composite were stable with good linearity and repeatability. The resistance change rate demonstrated high sensitivity to mechanical strain with gauge factors of 34–71. With the appearance of a crack, the resistance of the composite film changed abruptly. Taking advantage of this, the locations and formation times of cracks can be preliminarily determined. The flexible composite film can monitor the strain and crack of a concrete surface well, and it is expected to be used for regional monitoring using an integrated approach.

## Figures and Tables

**Table 1:** The resistivity and conductivity of the composite.

**RGO Content (wt%)**	0.4	0.8	1.2	1.6
**Resistivity (Ω·m)**	1.07 × 10^2^	8.74 × 10^0^	2.33 × 10^0^	9.25 × 10^−1^
**Conductivity (S/m)**	9.35 × 10^−3^	1.14 × 10^−1^	4.29 × 10^−1^	1.08 × 10^0^
